# Assessing inequalities and regional disparities in child nutrition outcomes in India using MANUSH – a more sensitive yardstick

**DOI:** 10.1186/s12939-020-01249-6

**Published:** 2020-08-13

**Authors:** Ayushi Jain, Satish B. Agnihotri

**Affiliations:** Centre for Technology Alternatives for Rural Areas (CTARA), Indian Institute of Technology, Bombay, Maharashtra 400076 India

**Keywords:** Composite index, Triple burden, Malnutrition, Balanced development, Spatial heterogeneity, Shortfall sensitivity, Hiatus sensitivity, Policy, India

## Abstract

**Background:**

India is strongly committed to reducing the burden of child malnutrition, which has remained a persistent concern. Findings from recent surveys indicate co-existence of child undernutrition, micronutrient deficiency and overweight/obesity, i.e. the triple burden of malnutrition among children below 5 years. While considerable efforts are being made to address this challenge, and several composite indices are being explored to inform policy actions, the methodology used for creating such indices, i.e., linear averaging, has its limitations. Briefly put, it could mask the uneven improvement across different indicators by discounting the ‘lagging’ indicators, and hence not incentivising a balanced improvement. Signifying negative implications on policy discourse for improved nutrition. To address this gap, we attempt to develop a composite index for estimating the triple burden of malnutrition in India, using a more sensitive measure, MANUSH.

**Methodology:**

Data from publicly available nation-wide surveys - National Family Health Survey (NFHS) and Comprehensive National Nutrition Survey (CNNS), was used for this study. First, we addressed the robustness of MANUSH method of composite indexing over conventional aggregation methods. Second, using MANUSH scores, we assessed the triple burden of malnutrition at the subnational level over different periods NHFS- 3(2005–06), NFHS-4 (2015–16) and CNNS (2106–18). Using mapping and spatial analysis tools, we assessed neighbourhood dependency and formation of clusters, within and across states.

**Result:**

MANUSH method scores over other aggregation measures that use linear aggregation or geometric mean. It does so by fulfilling additional conditions of Shortfall and Hiatus Sensitivity, implicitly penalising cases where the improvement in worst-off dimension is lesser than the improvement in best-off dimension, or where, even with an overall improvement in the composite index, the gap between different dimensions does not reduce. MANUSH scores helped in revealing the gaps in the improvement of nutrition outcomes among different indicators and, the rising inequalities within and across states and districts in India. Significant clusters (*p* < 0.05) of high burden and low burden districts were found, revealing geographical heterogeneities and sharp regional disparities. A MANUSH based index is useful in context-specific planning and prioritising different interventions, an approach advocated by the newly launched National Nutrition Mission in India.

**Conclusion:**

MANUSH based index emphasises balanced development in nutritional outcomes and is hence relevant for diverse and unevenly developing economy like India.

## Background

United Nations’ Sustainable Development Goals 2 and Goal 3 reaffirm that proper nutrition is central to well - being of all individuals [1]. Malnutrition, if left unattended in the early age of a child, results in an irreversible effect on the cognitive and physical development of an individual hindering the overall development of human capital and economic growth [2]. Malnutrition contributes to nearly 68% of deaths in children under 5 years of age in India in the year 2017 [3].

Recent surveys [4, 5] confirm that India is grappling with the triple burden of malnutrition among children under the age of five – undernutrition, and micronutrient deficiencies among many and emerging overweight/obesity in some. The coexistence all three forms of malnutrition are often linked to changes in the structure of food systems, economic transitions and income inequalities [6]. With explicit cognizance of the alarming scenario, nutrition has eventually taken centre stage of development agenda, in recent times, in India. A National Nutrition Strategy was formulated in the year 2017, by NITI Aayog, the think tank body of Government of India, to cater to the problem of child and maternal malnutrition in India through focussed planning and convergent approach [7]. This eventually led to the launch of National Nutrition Mission (NNM), also known as ‘POSHAN Abhiyaan’, in March 2018. Targets have been set to reduce the prevalence of under-weight and low birth weight in children below 6 years of age by 6% each (@2% per annum), anaemia among children, adolescent girls and women by 9% (@3% per annum) and to bring down stunting in children below 6 years of age to 25% by the year 2022 [8]. It may be noted that the problem of wasting and overweight/obesity has not been accorded priority even though its emergence needs to be recognised and tackled early [3].

The Abhiyaan or the campaign, has been adopted to create a nationwide societal movement for improved national outcomes through convergent action at state and district level. The rolling out of NNM in all districts across the country were done in a phased manner within duration of 2 years (2017–20), i.e. 315 districts in the first year (2017–18), 235 districts in the second year (2018–19) and remaining districts to be covered in the third year (2019–20) [8].

To measure and monitor the progress made at the national and sub-national level, efforts are being made to establish robust indicators, analytical tools and methodologies and enhanced use of mapping techniques and data visualisation tools to provide a clearer picture of the persisting problem of malnutrition – which in itself is multidimensional and complex and in nature. This is evident from a recent upsurge in the number of such indices to assess levels of development on different fronts, such as NITI Aayog’s Health Index [9], SDG index [10] and Food and Nutrition Security Analysis [11]. These efforts in India are by and large a reflection of the global discourse that has witnessed the development of similar indices [12–17] to assess levels of poor nutrition, hunger and food insecurity across different countries. These indexes aim to serve as a tool for policymakers and various stakeholders to provide information on the world’s ‘state of art’ information on nutrition and health. However, no standalone index has yet been developed that specifically addresses the problem of triple burden of malnutrition in India.

Use of such an index is critical especially in a country like India, as it can help decision makers to assess and analyse the non –uniform nature of development and inequalities in achieving improved nutrition across and within different regions at the national and sub-national level. This could eventually help in planning, allocating right resources and funds. The selection of right indicators, data quality, and method of aggregation used to create such composite indices thus becomes essential [18]. While there is considerable debate on the choice of indicators and data quality in the received literature, studies that focus on the selection of appropriate aggregation methods in creating composite indexes [19], are limited and need special attention.

Several aggregation methods have been proposed in the literature, all with their advantages and drawbacks, specific assumptions and properties. Linear aggregation method is the most common among these, extensively used in all the recent development indexes in India mentioned earlier in this paper. A problematic feature of this method is the masking effect due to the full substitutability among its components; higher values of some components can compensate for lower values of other components [19, 20]. Even Human Development Index (HDI), prior to 2010, was computed using linear aggregation method. It was noted for instance, that a weaker state of health in a particular country, could get masked in the aggregation by, say, improved income levels. Appropriateness of the linear aggregation method was as such debated and critiqued by several researchers and from 2010 onwards, computation of HDI shifted to the Geometric Mean approach [21, 22]. However, the geometric mean method too suffers from certain limitations and inadequacies, as noted by various researchers, including Mishra & Nathan (2018) [23].

Mishra & Nathan (2018) [23] have proposed a set of six conditions that a composite measure should satisfy – **M**onotonicity, **A**nonymity, **N**ormalisation, **U**niformity, **S**hortfall Sensitivity and **H**iatus Sensitivity using an α measure. The abbreviated set also referred to as ‘MANUSH’, addresses inequality among individuals or subgroups within each dimension, as well as inequality between and within different dimensions. Both the linear and geometric means satisfy the first three of these conditions viz. — **M**onotonicity, **A**nonymity and **N**ormalization. The geometric mean also satisfies an additional condition viz. **U**niformity. The MANUSH method satisfies, in addition, the other two requirements. The **S**hortfall sensitivity requires that the improvement in worse-off dimensions should be at least in proportion or greater than better-off dimension. The **H**iatus sensitivity requires that the higher overall attainment (where nature of indicator used is positive) or greater overall reduction (where nature of indicator used is negative) must simultaneously lead to a reduction in the gap across dimensions [23]. MANUSH method thus seems relevant and worth exploring for the purpose of our study which aims at developing an index that would put a premium on a balanced and even achievement in reduction of child malnutrition in the country.

This paper, thus, aims to achieve following three objectives. First, to develop a composite index to estimate triple burden of malnutrition in children under the age of five, in states and districts of India using MANUSH aggregation measure. Second, using mapping and spatial analysis techniques, project MANUSH scores to reveal inequalities at the national and subnational level, i.e. within and across states in India and compare the differences in three distinct time frames using data from surveys NFHS-3 (2005–06) [24], NFHS-4 (2015–16) [4] and CNNS (2016–18) [5]. Third, discuss the policy implications of using MANUSH for creating composite indexes, to support actions taken by policymakers, in order to combat malnutrition in India.

## Methodology

### Data and dimensions

The index for triple burden of malnutrition was computed for the respective states, union territories (UTs) and districts in India covered under three nation-wide cross-sectional surveys - National Family Health Survey-3 (2005–06) (NFHS-3) [25], National Family Health Survey-4 (2015–16) (NFHS-4) [26, 27] and Comprehensive National Nutrition Survey (2016–18) (CNNS) [28] respectively, conducted under the aegis of Ministry of Health and Family Welfare (MoHFW), Government of India. While International Institute for Population Sciences (IIPS), Mumbai is the nodal agency for conducting different rounds of National Family Health Survey, the Comprehensive National Nutrition Survey (CNNS) was conducted for the first time in India, led by UNICEF and Population Council, New Delhi. The ethics committee at the International Institute for Population Sciences, Mumbai in association with Ministry of Health, India assessed and approved the protocol of National Family Health Survey. Whereas, ethical approval for conducting CNNS was obtained from the Population Council’s Institutional Review Board (IRB) in New York, internationally, and from ethics committee of PGIMER in Chandigarh at national level, prior to initiating survey activities.

All the three surveys were chosen for comparison of malnutrition burden across States and UTs of India, over time, as they share similar characteristics with respect to the sampling design, reporting of common nutrition indicators and coverage of nationally representative sample. However, it is to note, that both NFHS-3 and CNNS were conducted only at the state level. Among union territories, only Delhi was surveyed. While, NFHS-4, not only covered all the 29 States and 7 Union Territories but also surveyed 640 districts in India. It is to be noted that Andhra Pradesh which was a composite state at the time of NFHS-3, got divided into two states – Andhra Pradesh and Telangana in the year 2014. More recently, in August 2019, the state of Jammu and Kashmir was divided into two union territories; since the analysis reported in this paper is based on the surveys conducted prior to 2019, we report findings for the composite state of Jammu and Kashmir.

To compute the index for the triple burden of malnutrition – undernutrition, micronutrient deficiency and overweight/obesity, a total of four component indicators were used in this study. Two indicators, namely, proportion of stunted children (in percentage) and proportion of wasted children (in percentage) under the age of five, were selected as indicators to characterize undernutrition. Proportion of anaemic children (in percentage) under the age of five was used as an indicator to describe micronutrient deficiency, especially that of iron. Overweight/ obesity was characterized by the proportion of children (in percentage) with weight for height z-score above + 2 standard deviation from the median of the reference population (set by the median of the WHO child growth standards). All the three surveys – NFHS-3, NFHS-4 and CNNS used the World Health Organisation (WHO) definitions [29, 30] for classifying children under the age of five as stunted, wasted, overweight/obese or anaemic. Children with height for age z-score and weight for height z-score below − 2 standard deviation from the median of the reference population (set by the median of the WHO child growth standards) were categorised as ‘stunted’ and ‘wasted’ respectively [29]. A child under the age of five was categorised as anaemic, if his/her haemoglobin levels was found to be less than 11.0 g per decilitre [30]. In all the three surveys, weight and height measurements of children under the age of five were carried out by trained health investigators using electronic digital weighing scale and a measuring board. For detection of anaemia, NFHS-3 [24] and NFHS-4 [4] deployed on site measurement of haemoglobin levels using a battery-operated portable HemoCue Hb 201+ analyser, by drawing a drop of blood by finger/heel pricking of children between 6 months and 59 months of age. Whereas, in CNNS [5], venous whole blood was drawn from children between 1 and 4 years of age, for assessment of anaemia based on haemoglobin concentration using the cyanmethemoglobin method. In all the three surveys, the haemoglobin levels were adjusted in areas with altitude levels above 1000 m.

Data for the four component indicators described above, reported as aggregate percentages, was obtained from the state and districts fact sheets, and compiled. Since the figures were taken from the government released fact-sheets for individual states and districts, no separate data cleaning was warranted.

### Standardisation of data

Values obtained for all the four selected indicators – Stunting, Wasting, Anemia, Overweight/Obesity were first standardized using a threshold value as a denominator. This threshold value was set nearest to the maximum observed value in the survey data for that indicator; hence they were set differently for states and districts. The standardized value was calculated as,

1 $$ {\mathrm{y}}_{\mathrm{ij}}={\mathrm{V}}_{\mathrm{ij}}/\mathrm{T} $$

where, y represents standardized value for a given component indicator for a particular state or district; i indicates the indicator, j represents either state or district, V_ij_ represents the actual value of given component indicator for a particular state or district, and T represents Threshold value. The threshold value for stunting, wasting, anemia and overweight/obesity was set at 60, 40, 85 and 10% respectively for states, and 70, 50, 95 and 20% respectively for calculating scores at the district level. Hence, the value of y_ij_ lied between 0 and 1. Lower the value of y_ij_, lower is the prevalence of stunted, wasted, anemic or overweight/obese children under the age of five.

### Assigning of weights

Next step involved assigning of weights to each of these standardized indicators. For this study, we assigned the weights (w_i_) of 1.7, 1.9, 1.0 and 1.7 respectively to the standardized values of stunting, wasting, anemia and overweight/obese respectively, as used in the study done by Swaminathan et al. (2019) [3].

### Calculation of index

After assigning weights, scores were computed for states and districts for estimating the triple burden of malnutrition using MANUSH aggregation measure. The MANUSH measure and the axiom definitions used by Mishra and Nathan (2018) [23] was modified for this study, as the indicators used in the present study were failure indicators and not attainment indicators, implying that in ‘ideal’ scenario, the value of the indicator should reduce to zero.

Thus, the triple burden index using MANUSH measure was derived as follows:

2 $$ {MANUSH}_{\propto }={\left[\frac{\sum \left\{{\left({w}_i{y}_{ij}\right)}^{\propto}\right\}}{\sum {w}_i^{\propto }}\right]}^{\left(1/\propto \right)} $$

where α = 1, 2, 3 …, …, ∞. The standardised higher values of α take the attainment pathways closer to the ideal path, which ultimately approaches the line of equity as α tends to infinity. In this paper, we have limited ourselves to the values of α = 2, which represents the displaced ideal method [23].

Interestingly when α = 1, the index takes the form of linear aggregation (LA) and thus derived as.

3 $$ LA=\left[\frac{\sum \left({w}_i{y}_{ij}\right)}{\sum {w}_i}\right] $$

On the other hand, geometric mean (GM) method for failure indicators take the form as one described below:

4 $$ GM=1-\left[\prod \left\{{\left(1-{y}_{ij}\right)}^{\left({w}_i\right)}\right\}\right] $$

Although, the focal point of the present study rests on MANUSH aggregation measure, composite scores for calculating triple burden were also computed using linear aggregation and geometric mean method, in order to highlight the difference between MANUSH and other two aggregation measures, wherever necessary. To bring out the differences in aggregation measures, the modified axioms were tested using districts as study unit, which is presented in detail in Additional File 2.

The value of each of these computed index scores ranged between 0 and 1. Since these are failure indicators, lower the score, the closer is the state to achieving an ideal state. The ranking of states and districts in India was done using the computed scores (See Additional File 1). All the calculations were done using STATA version 15 (StataCorp, College Station, TX, USA).

### Categorisation of states and districts on severity scale

For the purpose of this study, states and districts were further categorised on 5-point severity scale based on MANUSH scores. Cut-off was set for states and districts separately: Low (< 0.35 for states; < 0.20 for districts), moderate (≥0.35 and < 0.45 for states; ≥0.20 and < 0.35 for districts), serious (≥0.45 and < 0.55 for states, ≥0.35 and < 0.50 for districts), alarming (≥0.55 and < 0.65 for states; ≥0.50 and < 0.65 for districts) and extremely alarming (≥0.65 for both states and districts).

### Maps and charts for visualization

To examine the scenario of triple burden across states and districts in India based on MANUSH scores, visualization maps were developed using QGIS version 3.4.0. Visualization charts were prepared using MS-Excel to study fluctuations or deviations between two different survey periods or in the same round of survey. To study spatial dependency across districts in India in terms of triple burden of malnutrition, spatial analysis was performed. GeoDa version 1.14.0 was used to produce Univariate LISA cluster maps.

## Results

The scores and ranks as estimates of triple burden of malnutrition, computed for states and districts in the respective surveys can be found in Additional File 1.

### Comparing the nutrition scenario in India at a different level of aggregation

#### Nutrition scenario across state boundaries in India

**Fig. 1:**
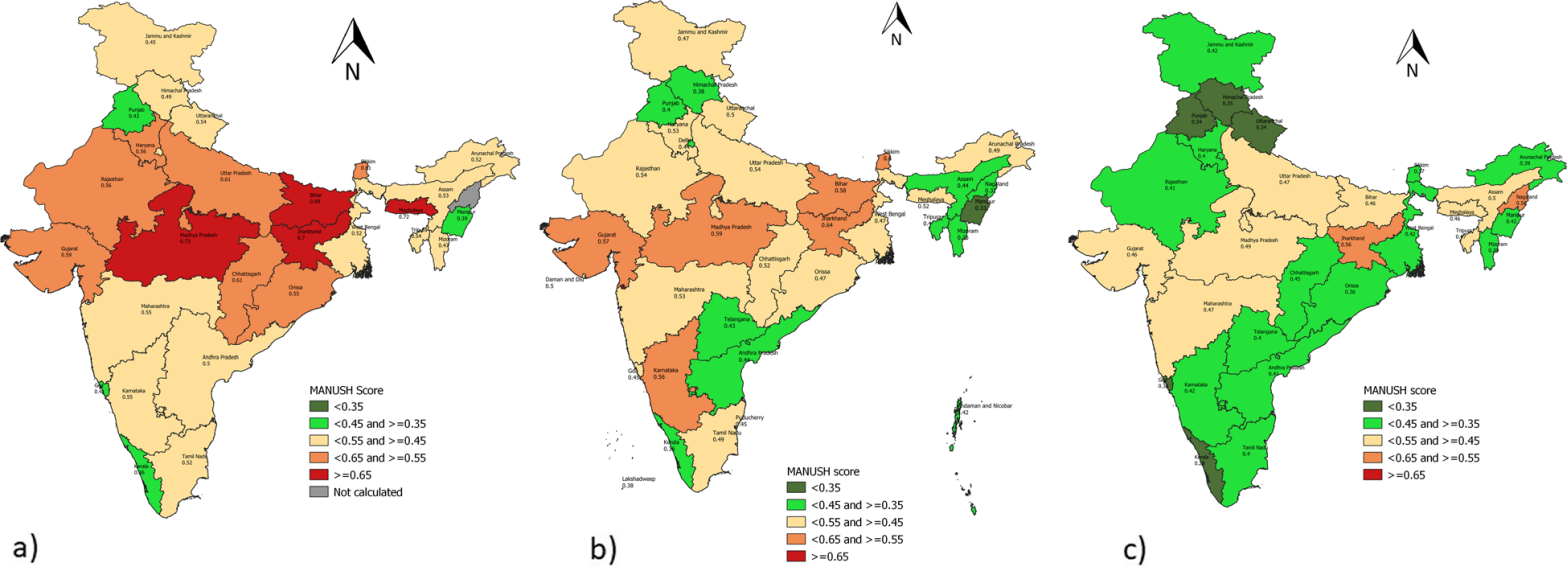
Map of India depicting state categorization on the severity scale based on MANUSH scores calculated based on **a** NFHS-3 **b** NFHS-4 **c** CNNS

Comparing the triple burden in states of India over three different periods of surveys, i.e. from NFHS-3 (2005–06) to NFHS-4 (2015–16) to CNNS (2016–18), a positive upward shift in the nutrition scenario at the state level is noticed, as seen in Fig. 1a-c.

Based on the MANUSH scores, at the time of NFHS-3 (see Fig. 1a), four states were found to fall under extremely alarming category (MANUSH score ≥ 0.65), namely Madhya Pradesh (0.73), Meghalaya (0.72), Jharkhand (0.70) and Bihar (0.69) on account of extremely high prevalence of stunted and wasted children below the age of five. Seven states – Uttar Pradesh (0.61), Sikkim (0.61), Chhattisgarh (0.61), Gujarat (0.59), Haryana (0.56), Rajasthan (0.56) and Odisha (0.55) were found in the alarming category as per MANUSH scores. All the seven states but Sikkim were penalized for the high prevalence of stunting, whereas Sikkim was accounted for the high prevalence of both stunted and overweight/obese children. Only four states – Punjab (0.43), Goa (0.41), Manipur (0.39) and Kerala (0.36) were found in the moderate category with MANUSH score in the range of ≥0.35 and < 0.45. Manipur (weighted standardized score wasting 0.07, anemia 0.08, overweight 0.06), when compared to Kerala (stunting 0.11, wasting 0.12, anemia 0.08, overweight 0.03) fared better in all dimensions, except that of stunting (weighted standardized score 0.16), and hence was penalized on account of unbalanced development across its dimensions. None of the states were found to fall under low category (MANUSH score < 0.35) while 13 states fell under the serious category (MANUSH score ≥ 0.45 and < 0.55). Nagaland was excluded from NFHS-3 study as the anemia prevalence was not known.

Next, the nutrition scenario in states surveyed in NFHS-4 (See Fig. 1b) was compared with previous round, i.e. NFHS-3 using MANUSH scores. In the NFHS-4 round, none of the states fell in the extremely alarming category (MANUSH score ≥ 0.65), except Dadra and Nagar Haveli (MANUSH Score 0.67), a Union Territory (UT) penalized on performing poorly on all four dimensions. Three states, Jharkhand (0.64), Madhya Pradesh (0.59) and Bihar (0.58), when compared to NFHS-3, moved from extremely alarming to alarming category on account of reduction in stunting and wasting, although overweight/obesity rose marginally in children below 5 years in these states with no or minimal reduction in anemia. Sikkim (0.60) and Gujarat (0.57) showed no change in the category on the severity scale between the two rounds and were retained in the alarming category. Karnataka (0.56), on the other hand, deteriorated its position, descending from serious to alarming category between NFHS-3 and NFHS-4. while Meghalaya (0.52) showed a significant upward transition from extremely alarming to serious category between two rounds of the survey. Uttar Pradesh (0.54), Rajasthan (0.54), Haryana (0.53), Chhattisgarh (0.52) and Odisha (0.47) demonstrated one -scale upward shift from alarming to the serious category in NFHS-4 on account of robust decline in stunting, followed by anemia. Except Odisha, wasting was found to increase in all the four states. Goa (0.45) slipped from moderate to serious category, penalized on account of an unprecedented rise in wasting and anemia, despite a small reduction in stunting and children under 5 years. While, Maharashtra (0.53), Uttarakhand (0.50), Tamil Nadu (0.49), Arunanchal Pradesh (0.49), West Bengal (0.47) and Jammu and Kashmir (0.47), were retained in serious category. Daman and Diu (0.50) and Puducherry (0.45), the union territories, are also found in serious category, with former penalized on account of a high prevalence of wasting and anemia and later due to high prevalence of wasting with respect to other dimensions. Assam (0.44), Delhi (0.44), Andhra Pradesh (0.44), Telangana (0.43), Tripura (0.40), Himachal Pradesh (0.38) and Mizoram (0.35) gradually shifted from serious to moderate category. Punjab (0.40) and Kerala (0.36) held in moderate category, showed no shift on the severity scale between the two rounds. Three UTs, namely, Andaman and Nicobar Island (0.42), Chandigarh (0.40), Lakshadweep (0.38) and Nagaland (0.37) also added to the list of states in the moderate category. Manipur (0.33) demonstrated a positive upward shift from moderate to low category and is the only state to be found in the low category.

**Fig. 2:**
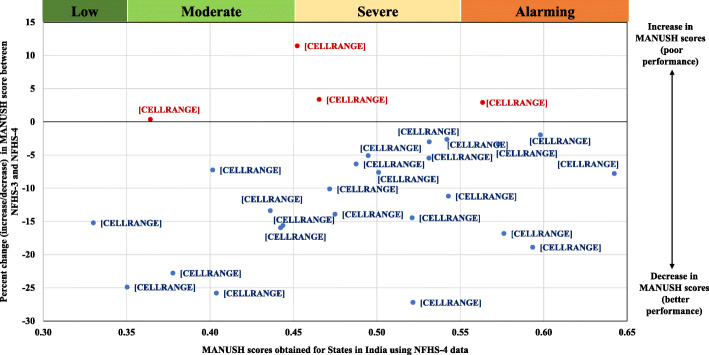
Percent reduction in MANUSH scores across states in India between NFHS-3 and NFHS-4 rounds of the survey

Except, Kerala, Jammu and Kashmir, Karnataka and Goa, all the states showed reduction in the MANUSH scores, between NFHS-3 and NFHS-4 (See Fig. 2). Meghalaya exhibited maximum improvement between the two rounds, followed by Tripura, Mizoram and Himachal Pradesh accounting to 23% or more reduction in MANUSH scores. Madhya Pradesh and Bihar, the two states accounting to the maximum burden of undernourished children, demonstrated reduction of 17–18% in MANUSH scores, suggesting that these states are progressing in the right direction in terms of addressing malnutrition, although steadily. Similar is the case of Delhi, Assam, Manipur, Chhattisgarh, Odisha, Andhra Pradesh, Uttar Pradesh and West Bengal that showed 10–15% reduction in MANUSH scores. States like Jharkhand, Uttarakhand, Punjab, Arunanchal Pradesh, Haryana and Tamil Nadu showed tardy reduction, of about 5–7%, between the two rounds. Gujarat, Maharashtra, Rajasthan and Sikkim showed the minimum decline (less than 5%), suggesting extremely marginal improvement between the two rounds. States like Kerala, Jammu and Kashmir and Karnataka, on the other hand, showed a marginal increase (≤5%) in MANUSH scores between two rounds, suggesting unbalanced development across the dimensions, and hence the penalty. Moreover, Goa showed increment of about 11% in MANUSH scores and has been heavily penalized on account of the unprecedented rise in wasting in children under the age of five.

Further comparison with recently released Comprehensive National Nutrition Survey (2016–18) conducted in 29 states of India and Delhi (Union Territory), a brighter picture of enhanced nutrition scenario across the states in India emerges at the surface (See Fig. 1c). No states were found to fall under extremely alarming category (MANUSH score > 0.65). Jharkhand continued to retain in alarming category (0.56), although it showed a modest reduction in MANUSH scores between NFHS-4 and CNNS. While Assam (0.50) and Tripura (0.47) fall from moderate to severe category, Nagaland (0.56) descends from moderate to alarming category. On the other hand, Madhya Pradesh (0.49), Gujarat (0.46) and Bihar (0.46) ascend from severe to moderate category. Maharashtra (0.47), Uttar Pradesh (0.47) and Meghalaya (0.46) were retained in a severe category despite decline in their MANUSH scores. A large chunk of states falls under moderate category during CNNS as per MANUSH scores. Sikkim (0.37) and Karnataka (0.42) moved dramatically upwards from alarming to moderate category. Eight states, namely, Chhattisgarh (0.45), Jammu & Kashmir (0.42), West Bengal (0.42), Rajasthan (0.41), Haryana (0.40), Tamil Nadu (0.40), Arunanchal Pradesh (0.39) and Odisha (0.36) showed an upward shift from severe to moderate category. Interestingly, Manipur (0.42), which ranked 1st in NFHS-4 survey dropped to 17th rank in CNNS, falling from low to moderate category, implying heavy penalty imposed by MANUSH indexing method on account of unbalanced development. Andhra Pradesh (0.41), Telangana (0.40), Mizoram (0.39) and Delhi (0.39) continued in moderate category. While Uttarakhand (0.34) and Goa (0.34) ascended from severe to low category, Himachal Pradesh (0.35), Punjab (0.34) and Kerala (0.28) also shifted from moderate to low category.

### Nutrition scenario across district boundaries in India

**Fig. 3:**
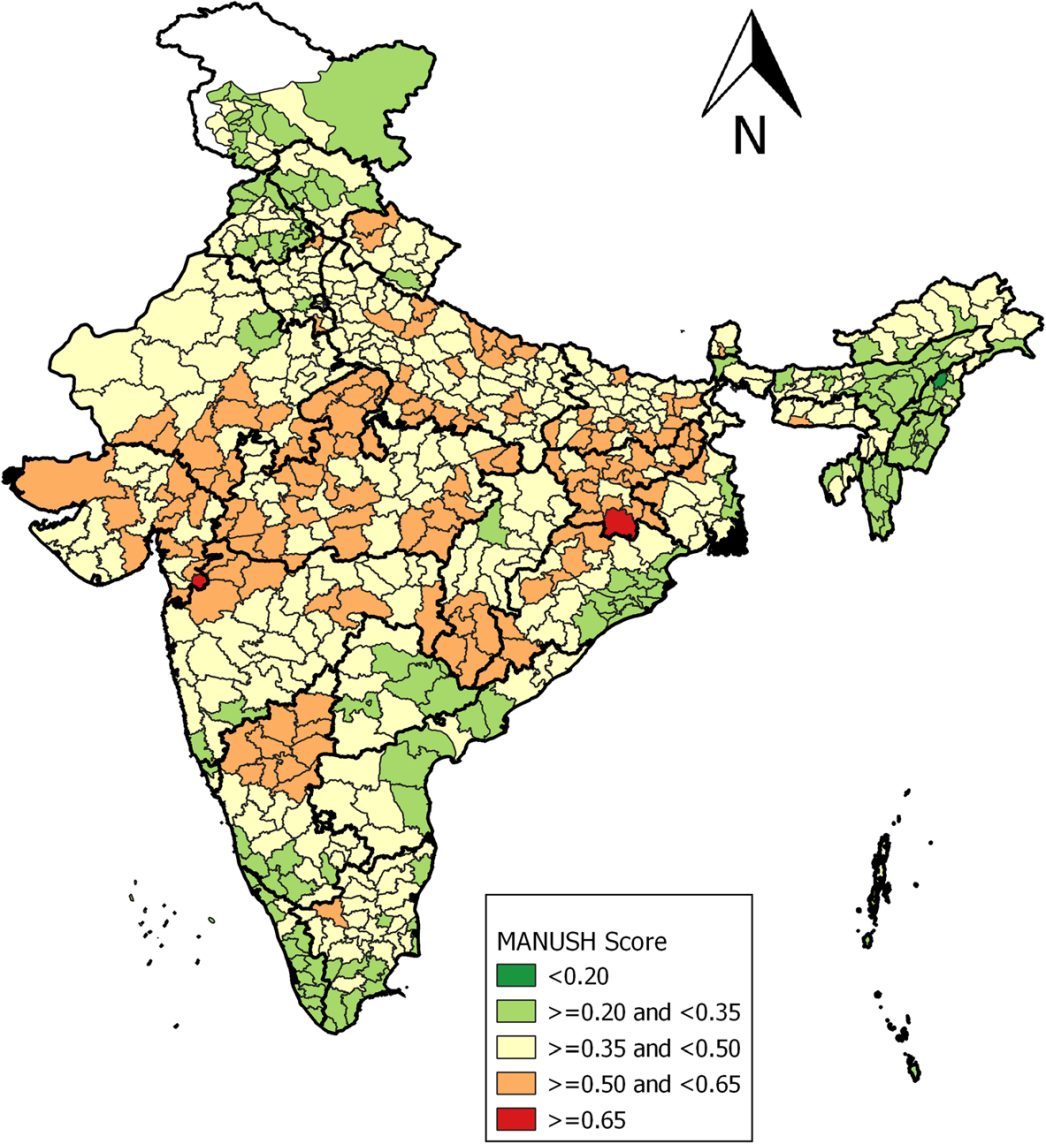
Map of India depicting district categorization on the severity scale based on MANUSH scores calculated using CNNS data

Next, the nutrition scenario across 640 districts of India was examined, based on MANUSH score and ranking (See Fig. 3) computed using district level data of NFHS-4.

Pockets of districts falling under extremely alarming, alarming, serious and moderate range emerge from the central and spread across the posterior end of the country in the said order. Two districts (Pashchimi Singhbhum of Jharkhand and The Dangs of Gujarat) fell in the extremely alarming category (score > 0.65), followed by 145 districts in the alarming category, a large proportion belonging to Madhya Pradesh, Uttar Pradesh, Jharkhand, Bihar, Rajasthan and Gujarat. Further, 352 and 140 districts fell in the serious and moderate category and only one district (Mokokchung district of Nagaland) was found in the low category of severity scale.

Of the districts falling in alarming and extremely alarming category (≥0.50 MANUSH score), i.e. a total of 147 districts, 40% were penalized on account of high incidences of wasting, about 30% were penalized due to high incidences of stunting and about 22% have higher prevalence of both stunting and wasting. Interestingly, Erode district in Tamil Nadu was severely penalized on account of high incidence of overweight/obese children under the age of five. Few other districts like Chennai of Tamil Nadu, Ambala in Haryana, Banda of Uttar Pradesh, South District of Sikkim, and Ambala district of Haryana have been penalized on account of overweight/obese children in addition to undernourished children. All the districts in the bottom two category have medium to a high prevalence of anaemic children under the age of five.

**Fig. 4:**
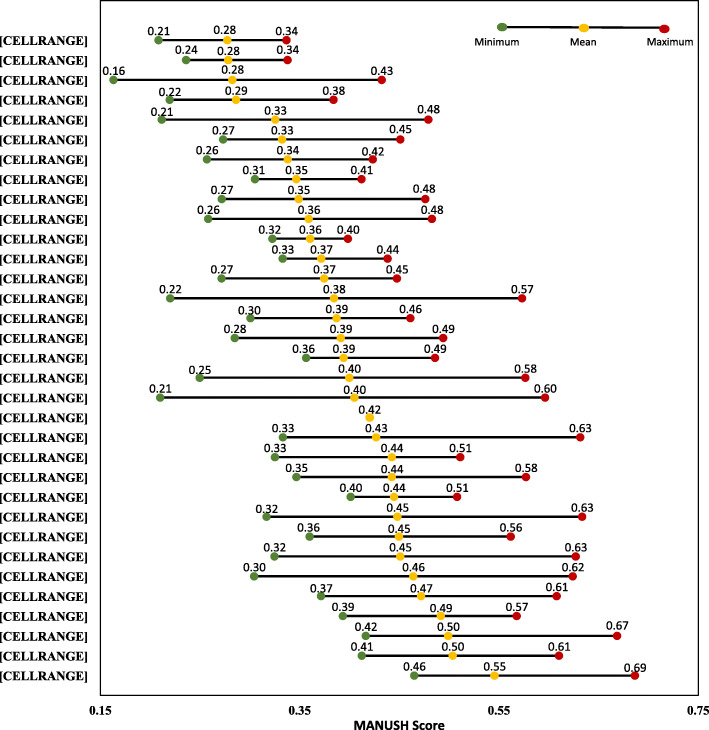
States arranged on the basis of moving average of MANUSH scores of districts. Note: On the y-axis, the value in brackets indicates the number of districts in that particular state

Figure 4 provides a snapshot of subnational disparities in triple burden of malnutrition across and within the States/UTs of India covered in NFHS-4 based on MANUSH scores. For each state, this figure shows the mean MANUSH score as well as the highest and lowest MANSUH score obtained by the districts in the state. Though, it is impossible to determine which states have the highest levels of inequality in terms of triple burden of malnutrition based solely on the size of the gaps between the highest and lowest subnational MANUSH scores, yet there are some interesting insights. For instance, in some states, the spread is quite compact, e.g. in Andhra Pradesh and quite large for some, e.g. Odisha and, surprisingly, even Tamil Nadu.

Interestingly, Odisha’s best performing district (Cuttack) with undernutrition score of 0.21, ranks 4th in the country while its worst-performing district, Nabarangpur, with a score of 0.60, ranks 623rd is amongst the worst-performing districts in the country. Similarly, Kanniyakumari of Tamil Nadu with a score of 0.22 ranks 7th in the country, while Chennai in Tamil Nadu ranks 611th and falls in the bottom list of worst-performing districts. Chennai shows higher prevalence in all the four indicators – overweight/obesity, stunting, wasting and anaemia in children under the age of 5 years. If one takes a closer look at the spread in the case of Odisha, the best and the worst-performing districts appear to form a cluster. A similar pattern is observed in West Bengal. In contrast to the case of Odisha and West Bengal, the states of Bihar and Madhya Pradesh each with 38 and 50 districts, have a low average MANSUH score for their state and a small deviation between the scores of their best performing and worst-performing districts, reflecting the uniformly poor performance across the state. Maharashtra, Karnataka and Rajasthan show a comparable spread. Such inequalities within and across states level reflect spatial heterogeneities that exist in India.

**Fig. 5:**
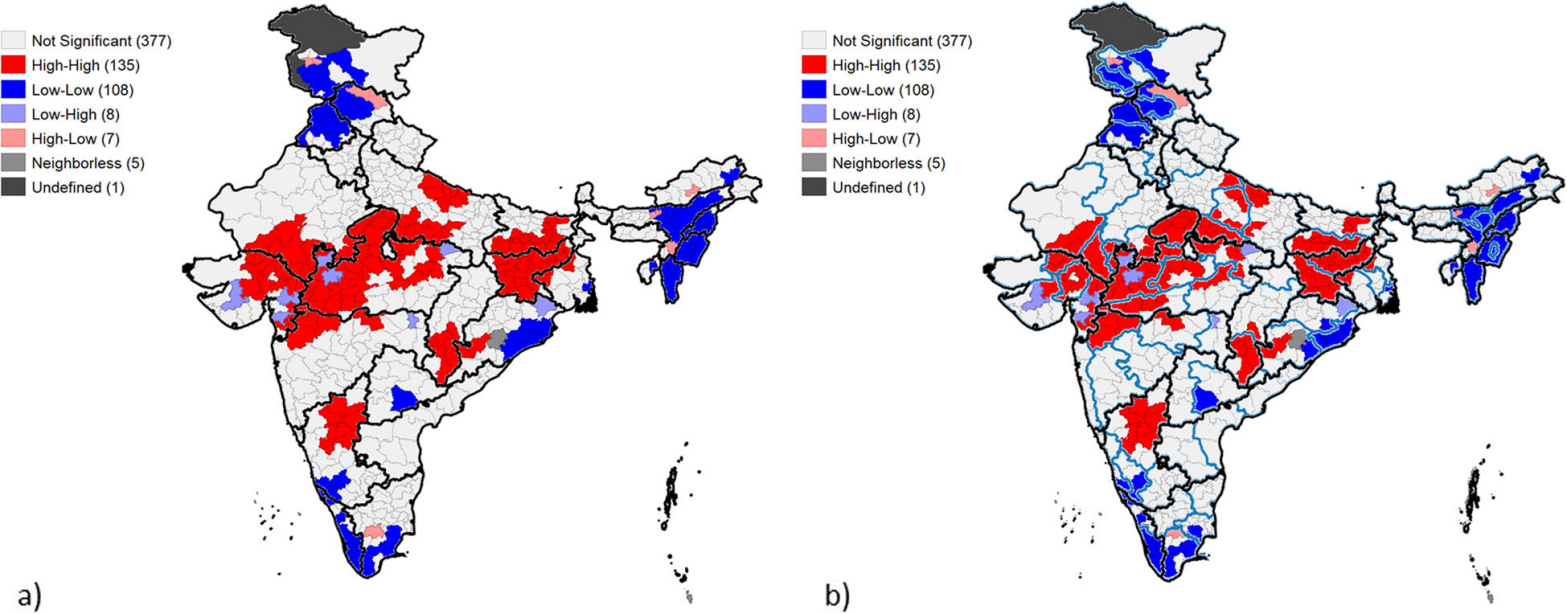
Univariate LISA cluster maps of India showing the geographic clustering based on MANUSH scores across districts of India, **a** with state boundaries, 2015–16. **b** with state and NSS boundaries (as per 68th round of NSS), 2015–16

To study the spatial heterogeneity at the district level, Univariate LISA maps were created using MANUSH scores using GeoDa version 1.14.0. As shown in Fig. 5a, formation of significant clusters (*p* < 0.05) was observed and the univariate Moran’s I value was found to be 0.619, depicting strong spatial autocorrelation. About 135 districts out of 640 districts, i.e. 20% were surrounded by districts with high MANUSH scores, signifying clusters of high malnutrition, while 108 districts, i.e. around 17% districts were surrounded by districts with low MANUSH scores, signifying better nutrition scenario. The high-high clusters were mostly found in the central belt of India, in the states of Madhya Pradesh, Jharkhand, Maharashtra, Bihar, Rajasthan, Uttar Pradesh, Gujarat and some parts of Chhattisgarh, Odisha, West Bengal and Karnataka. While low-low clusters were found in the peripheral states of India – primarily in the north-eastern states like Nagaland, Manipur, Mizoram, some parts of Assam, Arunanchal Pradesh and Tripura, also in the northern states like Jammu & Kashmir, Himachal Pradesh and Punjab, in the southern coastal belt of Karnataka, Kerala and Tamil Nadu and in eastern coastal belt of Odisha and West Bengal. Interestingly, low-low clusters are also found in few districts of Telangana, bordering Andhra Pradesh. Spatial analysis also brought two groups of outliers, i.e. districts with low MANUSH scores (eight in count) surrounded by districts with high MANUSH scores, that can be grouped under ‘positive outliers’, and second, districts with high MANUSH score (seven in count) surrounded by districts with low MANUSH scores, that can be grouped under ‘negative outliers’.

Also, when we study the spatial clustering at the National Sample Survey (NSS) region level, the differences within a state become much more evident. NSS regions are the ones used for survey by National Sample Survey Organisation (NSSO) in conducting surveys across India on various socio-economic aspects since 1950. These regions are formed by contiguous grouping districts within a state, in relatively homogenous regions based on geographical features, rural population densities and crop-pattern [31]. As observed in Fig. 5b, if one takes a closer look at the spread in the case of Odisha, the best and the worst-performing districts appear to form significant clusters at coastal and southern NSS regions respectively. Similarly, in Karnataka and West Bengal, pockets of low performing and better performing districts appear at the anterior and posterior end, respectively, that are separated by NSS boundaries. Moreover, in states like Chhattisgarh and Bihar, significant clusters of poor-performing districts seem confined to a particular NSS region. A similar scenario is observed in Maharashtra, Gujarat and Rajasthan, where pockets of poor-performing districts seem to grouped together not only in a particular NSS region but also form clusters, across state boundaries, suggesting the spatial dependency of neighbours on malnutrition.

### Implications of MANUSH indexing on nutrition policy

Scoring and ranking of states and district have become a common phenomenon in recent times, in India, for ease of comparison and prioritization of issues and for development of policies. However, there are two limitations that have been observed in this context. First, if scoring is based on a combination of indicators, linear averaging is the only method of aggregation used, whose limitations have already been discussed in the sections above. Second, if the scoring is done on single parameters, for example, only stunting or wasting or anaemia, and policy is developed based upon it, there is an increased risk of overlooking other indicators.

To understand this, we looked at an actual policy priority measure in the recently launched National Nutrition Mission (NNM) in India. While the Mission objective is to reduce undernutrition in children, adolescent girls and women and achieve targets set for the year 2022 [7], it chose to group districts into three phases, for accelerated intervention, in order of priority. However, the priority was set solely based on the prevalence of stunting. The districts with the highest level of stunting were placed in the list of Phase I districts and so on. If the only objective were to reduce stunting, then this prioritization would have seemed rational. But to reduce undernutrition holistically – stunting, wasting and anaemia, dimensions which are independent and not a substitute for each other, need to be taken together. Not considering other two dimensions, might have led to misallocation of priorities, funds and resources. To comprehend this better, we ranked the same 640 districts according to MANUSH scores and grouped into 3 priority regions – priority 1, 2 and 3. Districts with MANUSH score ≥ 0.48 were accorded 1st priority, districts with MANUSH score between 0.38 and 0.48 were placed in the 2nd priority list, and districts with MANUSH score less than 0.38 was put in 3rd priority list (see Additional File 1). It is to be noted that MANUSH index discussed in this article, also takes into account wasted and overweight children under the age of five, along with stunted and anaemic children. It needs to highlighted because targets set under the National Nutrition Mission, 2018 do not talk about the reduction in wasting and overweight/obese children, who are on the rise in India.

**Fig. 6:**
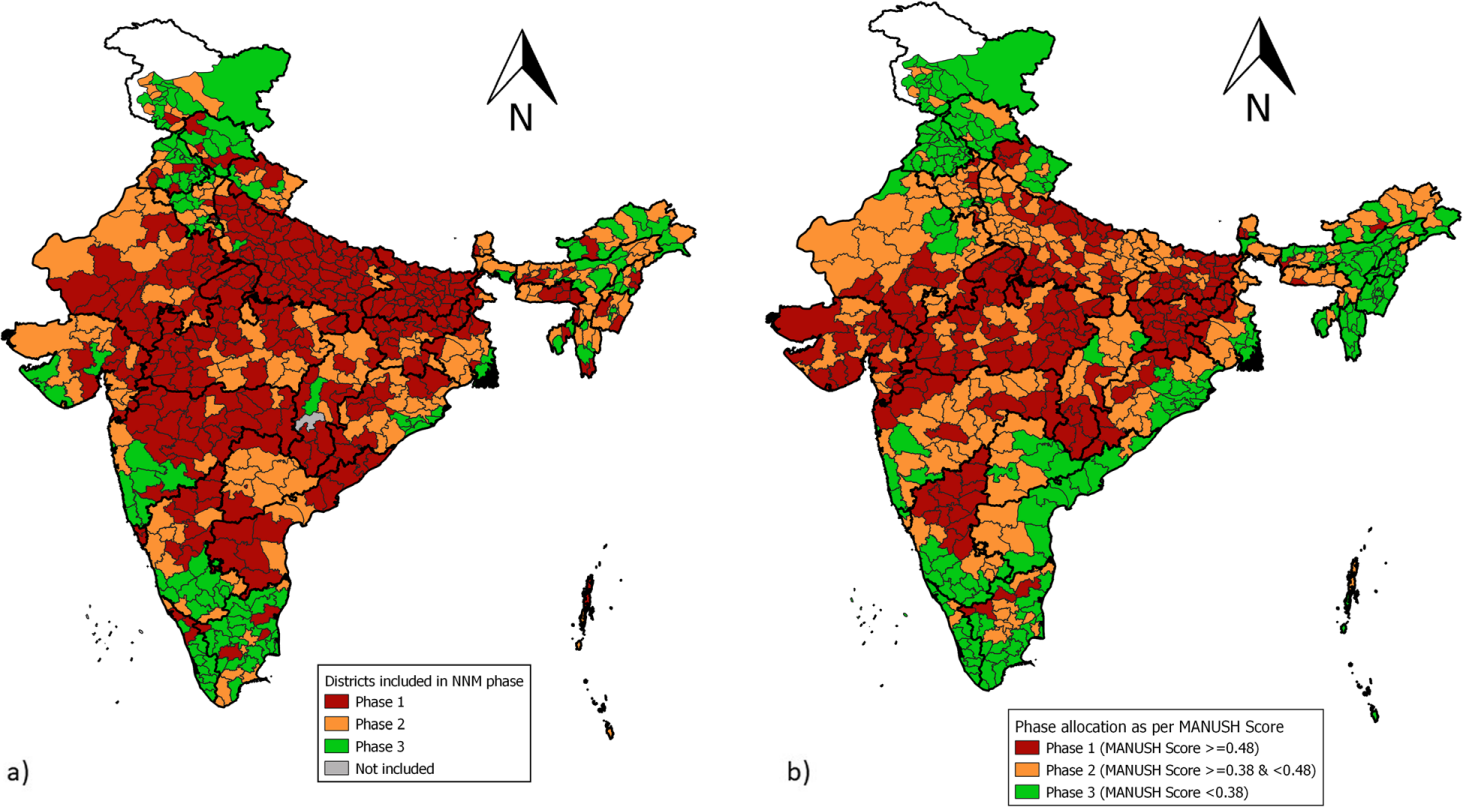
Map of India depicting phase allocation of districts as listed in **a** National Nutrition Mission and **b** as per MANUSH scores

After grouping the districts into priority 1, 2 and 3 regions, we compared these districts with the priority districts under National Nutrition Mission (NNM), rolled out in three phases, as shown in Fig. 6 below. About 8 districts (Dehradun, Ambala, Jamnagar, Chitradurga, Tiruvannamalai, Erode and Dharmapuri) which should have been part of phase 1 of NNM round as per MANUSH ranking, are listed in the 3rd phase of NNM. All the eight districts but Erode have been penalised on account of a high prevalence of wasting. Erode district of Tamil Nadu, on the other hand, is penalised on account of a high prevalence of overweight children, followed by stunted, wasted and anaemic children. If we focussed solely on indicators of undernutrition, then this district would have been neglected, but taking overweight as a measure in MANUSH index, it pinpoints those districts as well who are becoming emerging capital of overnutrition, alongside undernutrition.

Similarly, 37 other districts, that should have been in phase 1 of NNM as per MANUSH ranking are part of the NNM 2nd phase. Majority of these districts too are penalised on the high prevalence of wasting, except South District of Sikkim and Mahe district of Pondicherry, who show a high prevalence of overweight/ obese children under the age of five.

Moving further, 33 districts which should have been part of phase 2 of NNM as per MANUSH ranking are found in the list of districts under NNM 3rd phase. Three districts (Faridabad, Rewari and Kolkata) out of 33 mentioned have been penalised on account of the high prevalence of anaemia while two districts (Tawang of Arunanchal Pradesh and Namakkal of Tamil Nadu) have been penalised on account high incidence of overweight/obese children, while the rest of the districts exhibit high incidences of wasting. Interestingly, Mahamayanagar of Uttar Pradesh, one of the districts among the 33 mentioned above, exhibits a very high burden of stunting, and lower incidence of wasting, anaemia and overweight/obese. It has been penalised by MANUSH on account of unbalanced development across the dimension.

## Discussion

This paper focused on achieving three broad objectives. First objective dealt with developing a composite index to estimate triple burden of malnutrition in children under the age of five, in states and districts of India using MANUSH aggregation measure. Although our study employs a new indexing methodology – MANUSH, to estimate triple burden, a similar kind of composite index, known as Global Nutrition Index (GNI) has been attempted in past by Rosenbloom et al. (2008) [32], to assess triple burden of malnutrition across countries, using linear aggregation method. This index was recently modified by Peng and Berry (2018) [33], who adopted the new HDI method, i.e. geometric mean to compute GNI. Similar attempts have been made by few other researchers to develop composite index to measure different facets of malnutrition and hunger across geographies, such as International Nutrition Index [34], now commonly known as the Global Hunger Index [12], the Global Hidden Hunger Index [35] and Net State of Nutrition Index (NeSNI) [36], using either linear aggregation or geometric mean method. The MANUSH indexing measure, as explained by [23], is more effective in bringing out inequalities across dimensions on account of fulfilling two additional axiomatic conditions – shortfall sensitivity and hiatus sensitivity to level, conditions not satisfied by Linear aggregation and Geometric mean aggregation measures. Briefly put, MANUSH indexing method implicitly penalises cases where the improvement in worst-off dimension is lesser than the improvement in best-off dimension, or where, even with an overall improvement in the composite index, the gap between different dimensions does not reduce. Thus, due to the difference in satisfying two additional conditions, we observe the difference in scores and thus in the ranking of states/districts using MANUSH method (for details see Additional File 1 and 2). It is important to note that MANUSH measure α (α ≥2) was modified in our study, as the dimensions represented child growth failures, and not attainment.

The second objective focussed on projecting MANUSH scores to reveal inequalities at the national and subnational level, i.e. within and across states in India and compare the differences in three distinct time frames using data from surveys NFHS-3 (2005–06), NFHS-4 (2015–16) and CNNS (2016–18) through application of mapping and spatial analysis techniques. At national level, on examining the change in MANUSH scores in states across any two survey rounds, i.e. between NFHS-3 and NFHS-4, and, NFHS-4 and CNNS, reduction in MANUSH scores of varying amounts was noticed in all states except in Goa, Jammu & Kashmir, Karnataka and Kerala between NFHS-3 and NFHS-4 and in Manipur, Tripura, Mizoram and Assam between NFHS-4 and CNNS. Though at the surface, substantial reduction in MANUSH scores observed in the states from 2005 to 2018 in India suggest considerable improvement in nutrition status of children under the age of five, deeper investigation reveals inequality in reduction across four dimensions in states during individual surveys.

For instance, all states reported under CNNS were penalised by MANUSH on account of either non-uniform development, less or disproportionate development of worse-off dimension or even with overall reduction in MANSUH scores, failure to reduce the gap across its dimensions. Highest penalty was imposed on Manipur, on account of high proportion of overweight/obese children followed by stunted children on side and low proportion of wasted and anaemic children on the other side. Although Karnataka and Kerala, showed a quarter reduction in MANUSH scores between NFHS-4 and CNNS, yet they were penalised by MANUSH on account of neglecting stunting and wasting, while effectively addressing anaemia and overweight/ obesity. Jharkhand and Chhattisgarh which show steady reduction in MANUSH scores between NHFS-4 and CNNS are also penalised on same account. Similarly, states of Manipur and Mizoram which ranked 1 and 2 respectively during NFHS-4, were penalised for not reducing the gap across its dimensions (stunting and overweight/obesity vis a vis wasting and anaemia), despite securing low MANUSH score. Chhattisgarh and Odisha are penalised on account of slow reduction in stunting and wasting over speedy reduction anaemia and overweight/obesity. While Maharashtra is penalised for neglecting wasting followed by stunting, Assam on the other hand is penalised for high stunting followed by wasting prevalence. Goa, however, is penalised for very slow reduction in wasting over other dimensions. It is interesting to note, unlike CNNS, few states in NFHS-4 such as Tripura, Uttarakhand, Tamil Nadu, Arunanchal Pradesh, Jammu and Kashmir and Kerala, have been rewarded on account of balanced development across its dimensions. This could be a result of continuous efforts directed by the state agency in addressing malnutrition, for instance better implementation of programs such as the Integrated Child Development Scheme and Rural Health Mission in Tamil Nadu and history of interventions in the social sector in Kerala [37]. Use of MANSUH score is thus advantageous as it puts emphasis on the lagging indicator. Penalty and reward received by states were indicated by high and low scores of MANUSH respectively, over Linear Aggregation and Geometric Mean scores. Thus, state level monitoring of triple burden of malnutrition using MANUSH scores reveal substantial variations in the prevalence for each of the malnutrition indicators, which can serve as a reference to study trends over time. A recent study [3] examined such state level variation and trends in key malnutrition indicators in India from 1990 to 2017 to inform subnational action in order to achieve the Indian 2022 and the global 2030 targets.

Next, from the district level analysis using MANUSH scores, we looked into subnational disparities existing within and across states of India. Data from NFHS-4 was used in the study. Geographical heterogeneities were observed, and inequalities were reflected both within and across states. These variations in the geography were confirmed using spatial analysis. The spatial maps revealed the formation of significant clusters of high burden districts and low burden districts. The disparity seems to magnify at a more granular level of geographical region such as NSS regions. Equally interestingly, this effect cuts across state boundaries. The analysis at the NSSO region is a new feature in the analysis of nutritional status. These regions occupy intermediate level of aggregation between a district and a state and inter – region variation within the same state is an important feature to be noted. Few recent studies [38–41] have explored spatial clustering of key malnutrition indicators such as stunting, anaemia, low birth weight, etc. and its correlates in India. However, use of composite scores like MANUSH, rather than singular estimates, in revealing spatial heterogeneities and regional disparities is more advantageous in terms of informing policy and strategic planning. The MANUSH scores for districts can further be used to study correlates of triple burden of malnutrition, which is not in the scope of present study.

Moreover, the robustness of MANUSH over other aggregation measures in terms of “Shortfall Sensitivity” and “Hiatus Sensitivity to level” is effectively illustrated using districts as examples (See Additional File 2). For instance, taking the case of Upper Siang in Arunanchal Pradesh and Kollam in Kerala, where wasting represents the worse-off dimension and anemia represents the best-off dimension. Kollam when compared to Upper Siang, shows a lower reduction, in the worse off dimension, i.e. in wasting, when compared to anemia, it’s best off dimension, and hence is penalized by MANUSH measure (α ≥2). We even notice that when we increase α measure, that is α = 3, 5 and 10, Kollam is further penalized on account of disproportionate reduction in its best -off and worse-off dimension. Conversely, taking another set of example, like of Dakshin Dinajpur of West Bengal and Kanniyakumari of Tamil Nadu, which follow the same order of indicator values, where, stunting represents the worse off dimension, followed by anaemia, wasting and overweight/obesity, represents the best-off dimension, we see that Kanniyakumari when compared to Dakshin Dinajpur, shows more reduction in its worse-off dimension, i.e. stunting, in comparison to its better off dimensions, it is not penalized by MANUSH measure (α ≥2), on account of being sensitive to shortfall across its best-off and worse-off dimension.

MANUSH satisfies additional condition of Hiatus sensitivity to level, as explained further. For instance, Surendranagar in Gujarat, has standardized values of stunting, wasting, anaemia and overweight/obesity as 0.18, 0.17, 0.13 and 0.03, respectively, and Bhojpur of Bihar has standardized values of stunting, wasting, anaemia and overweight/obesity as 0.17, 0.16, 0.12 and 0.02, respectively, showcasing same gap across dimension, though, Bhojpur is moving towards greater overall reduction. Hence, MANUSH penalizes Bhojpur for having same gap across dimension, a phenomenon not elucidated by linear aggregation or geometric mean.

MANUSH has been previously used by [42] to evaluate Human Development Index for 127 countries between the years 1990 and 2004. It has also been employed by [43] to assess the decline in child undernutrition and socio-economic disparities in India between 2005 and 06 and 2015–16. Human development in Odisha was studied by [44] who performed inter district analysis using MANUSH.

Lastly, we studied the implication of ranking districts using MANUSH score for policy development using phasing of districts under the National Nutrition Mission as an example. The findings reveal that interventions targeted towards addressing malnutrition in India must get away with ‘One size fit all’ approach, instead develop context specific action plans, focussing on districts or NSS regions, which have proven to be more homogenous in nature. MANUSH indexing method is an appropriate tool for creating indices to estimate the burden of malnutrition at a different level of aggregation, taking different dimensions into account, bringing out the inequalities and gaps and hence can serve a sound decision support system. This methodology can thus be applied to other contexts as well where the development is sought to be equitable and balanced.

## Conclusion

To sum up, this study is thus important on three fronts – first, it assesses the triple burden of malnutrition (undernutrition, overweight/obesity and anaemia), including its spatial dimension, using a composite index for the first time. Methodologically, it uses MANUSH in developing an index on malnutrition, using failure indicators, for the first time. Third, a comparative assessment of nutrition scenario at state level between three periods NHFS-3 (2005–06), NFHS-4 (2015–16), CNNS (2016–18) data and spatial analysis to study the pattern of triple burden at districts and the National Sample Survey (NSS) region level using NFHS-4 data, is a new contribution to existing literature. Disparities across different NSSO regions within the same state is an interesting and important feature of this analysis.

Composite Indexing, thus, reflects better, the burden and heterogeneities using spatial maps, which is difficult using single parameters. Thus, MANUSH indexing becomes important on various fronts. First, it allows us to aggregate different dimensions together, taking care of the shortfall and hiatus across and within dimensions, thus prioritizing balanced improvement. Second, use of MANUSH scores, facilitates better understanding of not only the actual burden in the district and its status compared to other, in addition, it also points out which dimension needs further attention and to what extent the reduction is needed and thus can be helpful in prioritizing the interventions. This promotes decentralized and context-specific planning and programme implementation. One of the common complaints about the data on child nutritional levels has been its quality and frequency. With the availability of the NFHS-4 (2015–16) data right at the district level, we have a relatively up to date good quality baseline that could inform the National Nutrition Mission. CNNS (2016–18) followed it closely though it is available only at the state level. Fortunately, the NFHS – 5 data is likely to be available in the near future, addressing the frequency issue. Use of MANUSH methodology for comparing the nutritional outcomes between NFHS-4 and NFHS-5 at the state, region and the district levels is likely to give valuable insights – we await this eagerly.

## Supplementary information

**Additional file 1.** Consists of 9 worksheets. NFHS-3 scores & ranks (State). Calculation and Tabulation for computing Linear Aggregation (LA), Geometric Mean (GM) and MANUSH scores and Ranks using NFHS-3 data for States in India, 2005–06. NFHS-4 scores & ranks (State). Calculation and Tabulation for computing Linear Aggregation (LA), Geometric Mean (GM) and MANUSH scores and Ranks using NFHS-4 data for States in India, 2015–16. CNNS scores & ranks (State). Calculation and Tabulation for computing Linear Aggregation (LA), Geometric Mean (GM) and MANUSH scores and Ranks using CNNS data for States in India, 2016–18. NFHS-4 scores & ranks (Districts). Calculation and Tabulation for computing Linear Aggregation (LA), Geometric Mean (GM) and MANUSH scores and Ranks using NFHS-4 data for Districts in India, 2016–18. Monotonicity Cases. Examples explaining Monotonicity axiom. Uniformity Cases. Examples explaining Uniformity axiom. Shortfall Sensitivity Cases. Examples explaining Shortfall Sensitivity axiom. Hiatus Sensitivity Cases. Examples explaining Hiatus Sensitivity to Level. Districts under NNM and MANUSH. List and ranking of districts phased under National Nutrition Mission (NNM) and its priority categorisation based on MANUSH scores.

**Additional file 2.** Axioms Tests (Monotonicity, Anonymity, Normalisation, Uniformity, Shortfall Sensitivity and Hiatus Sensitivity to Level).

## Data Availability

Data used in this study for analysis is publicly available and can be obtained from http://rchiips.org/NFHS/index.shtml and https://nhm.gov.in/index1.php?lang=1&level=2&sublinkid=1332&lid=713.

## Abbreviations

MANUSH: Monotonicity Anonymity Normalisation Uniformity Shortfall Sensitivity Hiatus Sensitivity to level
SDGs: Sustainable Development Goals
NFHS: National Family Health Survey
CNNS: Comprehensive National Nutrition Survey
HDI: Human Development Index
UTs: Union Territories
WHO: World Health Organisation
LA: Linear Aggregation
GM: Geometric Mean
