# Correction to: Assessing inequalities and regional disparities in child nutrition outcomes in India using MANUSH – a more sensitive yardstick

**DOI:** 10.1186/s12939-020-01282-5

**Published:** 2020-10-07

**Authors:** Ayushi Jain, Satish B. Agnihotri

**Affiliations:** Technology, Bombay, Maharashtra 400076 India

**Correction to: Int J Equity Health (2020) 19:138**

**https://doi.org/10.1186/s12939-020-01249-6**

Following publication of the original article [1], the authors identified an error in Figs. 2 and 4. The correct figures are given below.

**Fig. 2:**
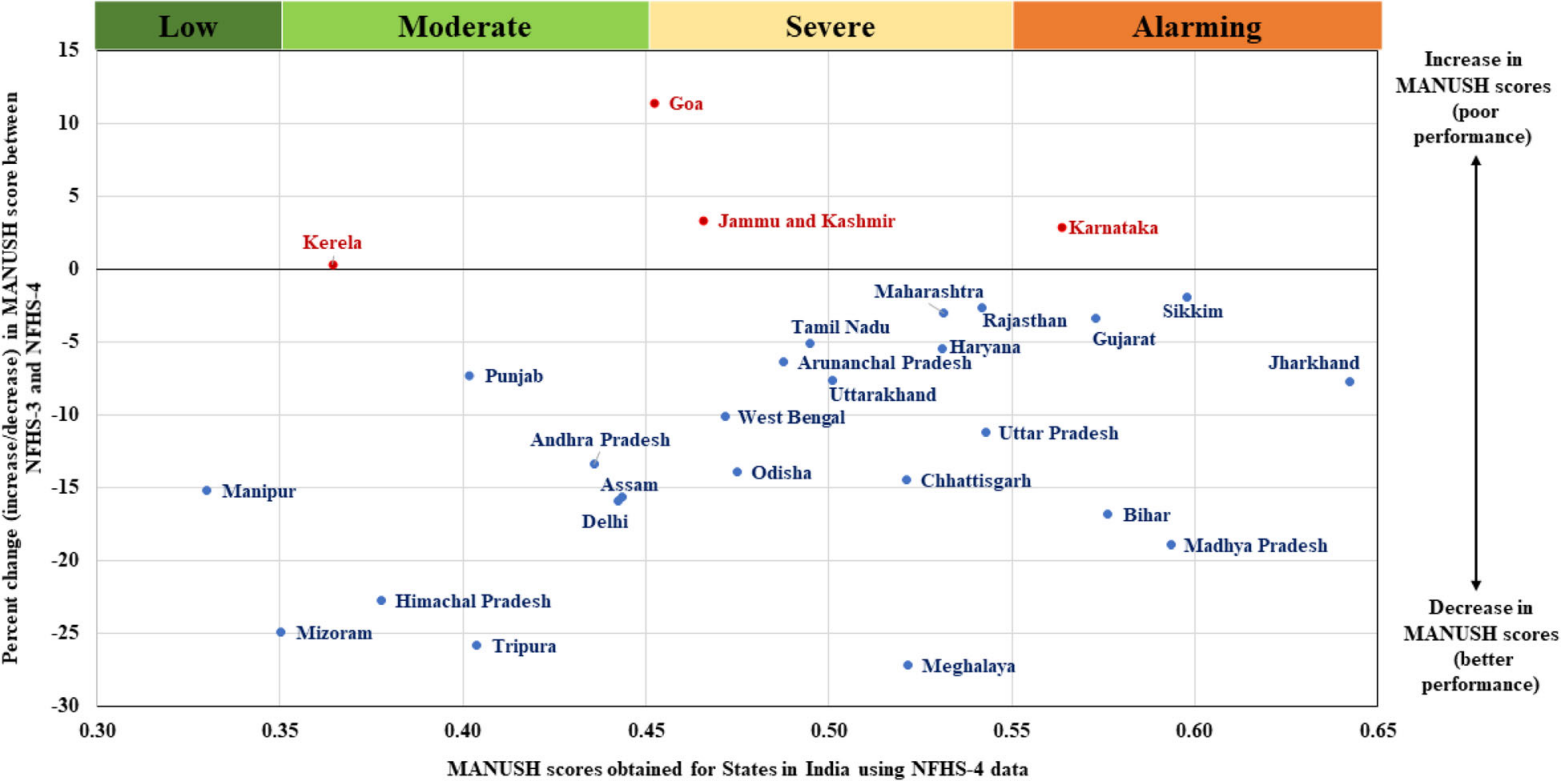
Percent reduction in MANUSH scores across states in India between NFHS-3 and NFHS-4 rounds of the survey

**Fig. 4:**
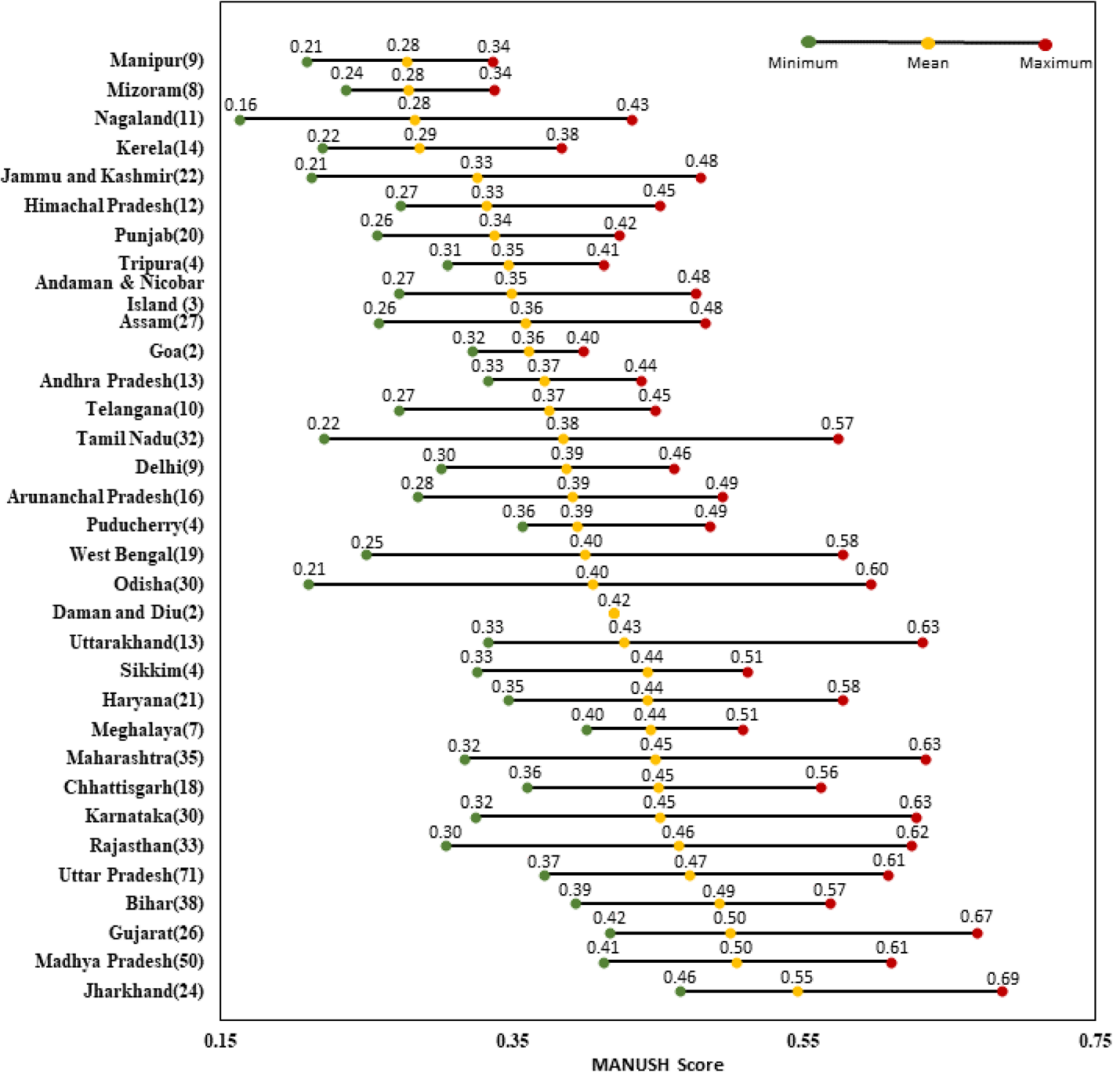
States arranged on the basis of moving average of MANUSH scores of districts. Note: On the y-axis, the value in brackets indicates the number of districts in that particular state
