# Supplementary material for: Assessing inequalities and regional disparities in child nutrition outcomes in India using MANUSH – a more sensitive yardstick
Source: Int J Equity Health. 2020 Aug 13;19:138. doi: 10.1186/s12939-020-01249-6 (PMC7427294; doi:10.1186/s12939-020-01249-6)
Supplement: Supplementary file 2 — Additional file 2. Axioms Tests (Monotonicity, Anonymity, Normalisation, Uniformity, Shortfall Sensitivity and Hiatus Sensitivity to Level). [file 12939_2020_1249_MOESM2_ESM.docx]

Additional File 2

**Axioms Test**

We first compared Linear aggregation and Geometric Mean method against MANUSH axioms, viz, Monotonicity, Anonymity, Normalization, Uniformity, Shortfall sensitivity and Hiatus sensitivity to level to bring out the differences in scoring patterns, if any, using districts as study unit.

It is to be noted that in the examples presented below, we have limited the values of individual indicators up to two decimals (except in case of normalisation axiom and shortfall sensitivity where these are limited to one and three decimal points respectively) to help in explaining our axioms, but cases may differ if we take the values to the third and subsequent decimal points. Hence, the caveat.

**Monotonicity.** A measure of aggregation (LA, GM or MANUSH) should be such that a decrease (increase) in failure in any one of the dimensions, other dimensions remaining constant, will lead to a decrease (increase) in the value of LA, GM or MANUSH.

For instance, given weighted standardized values of stunting, anaemia and overweight/obese being constant if standardized value of wasting is 0.13 in one district (case #1: example, Kaimur (Bhabua) in Bihar) and 0.09 in another (case #2: example, Vaishali in Bihar) then aggregation measure in case #1 should be greater than case #2.

Table 1: Cases explaining Monotonicity axiom

|  |  |  | **Weighted Standardized values** | | | | **Aggregation Scores** | | |
| --- | --- | --- | --- | --- | --- | --- | --- | --- | --- |
| **Case** | **District** | **State** | **wy_ST_** | **wy_WA_** | **wy_AN_** | **wy_OW_** | **GM** | **LA** | **MANUSH  (α = 2)** |
| #1 | Kaimur (Bhabua) | Bihar | 0.21 | 0.13 | 0.11 | 0.01 | 0.53 | 0.45 | 0.52 |
| #2 | Vaishali | Bihar | 0.21 | 0.09 | 0.11 | 0.01 | 0.51 | 0.42 | 0.49 |

We see from the above example that all the three indexing methods – linear, geometric and MANUSH satisfy the monotonicity condition. It is also to be noted that the geometric mean method will violate monotonicity if one of the dimensions has a value unity (1-unity being zero) and the other dimension keeps decreasing. Other instances where the condition of monotonicity is being fulfilled is presented in Additional File 1.

**Anonymity.** *A measure of aggregation (LA, GM or MANUSH) should be invariant to the weight-adjusted interchange of failure level across dimensions.*

For instance, given weighted standardized values of wasting and overweight/obese, if weighted standardized values of stunting and anaemia are 0.09 and 0.08, respectively, in case #1 and 0.08 and 0.09, respectively, in case #2 then value of aggregation measure in case #1 should be equal to value case #2, as seen in the case of Sangli district of Maharashtra and Sivaganga district of Tamil Nadu below.

Table 2: Cases explaining Anonymity axiom

|  |  |  | **Weighted Standardized values** | | | | **Aggregation Scores** | | |
| --- | --- | --- | --- | --- | --- | --- | --- | --- | --- |
| **Case** | **District** | **State** | **wy_ST_** | **wy_WA_** | **wy_AN_** | **wy_OW_** | **GM** | **LA** | **MANUSH  (α = 2)** |
| #1 | Sangli | Maharashtra | 0.09 | 0.11 | 0.08 | 0.01 | 0.31 | 0.29 | 0.32 |
| #2 | Sivaganga | Tamil Nadu | 0.08 | 0.11 | 0.09 | 0.01 | 0.31 | 0.29 | 0.32 |

All the three indexing methods - linear, geometric and MANUSH satisfy Anonymity condition.

**Normalization.** *A measure of aggregation (LA, GM or MANUSH) should lie between zero and unity such that at zero all dimensions have no failure (the ideal situation, the value of aggregation measure =0) and at unity, all dimensions have their maximum failure, the value of aggregation measure =1).*

Table 3: Cases explaining Normalization axiom

|  |  |  | **Standardized values** | | | | **Aggregation Scores** | | |
| --- | --- | --- | --- | --- | --- | --- | --- | --- | --- |
| **Case** | **District** | **State** | **y_ST_** | **y_WA_** | **y_AN_** | **y_OW_** | **GM** | **LA** | **MANUSH  (α = 2)** |
| #1 | Lower Subansiri | Arunanchal Pradesh | 0.5 | 0.5 | 0.5 | 0.5 | 0.5 | 0.5 | 0.5 |
| #2 | Cuttack | Odisha | 0.2 | 0.2 | 0.2 | 0.2 | 0.2 | 0.2 | 0.2 |

All the three indexing measures - linear, geometric and MANUSH follow the condition of normalization as seen in the case above. It is implicit that if all dimensions have a common value then aggregation measure (LA, GM or MANUSH) will have that common value and if the common value is zero or unity, respectively then LA, GM or MANUSH will be zero or unity, depicting no failure or maximum failure respectively. Identifying the cases that explicitly satisfy the condition of normalization, was difficult in the present data set. However, reducing the decimal digits to one, we have identified few cases such as of Lower Subansiri district in Arunanchal Pradesh and Cuttack district in Odisha that prove the aforementioned axiom.

**Uniformity.** *A measure of aggregation (LA, GM or MANUSH) should be such that for a given weighted-average of failure, a greater deviation across dimensions should give a greater aggregation value.*

For instance, for a given weighted standardised values of stunting and anaemia, if weighted standardised values of wasting and overweight/obesity are 0.19 and 0.01, respectively, in case #1 (example, Dhar district in Madhya Pradesh) and 0.12 and 0.08, respectively, in case #2 (example, Karnal district in Haryana), then value of aggregation measures in case #1 should be greater than in case #2.

Table 4: Cases explaining Uniformity axiom

|  |  |  | **Weighted Standardized values** | | | | **Weighted Average** | **Deviation from Weighted average** | **Aggregation Scores** | | |
| --- | --- | --- | --- | --- | --- | --- | --- | --- | --- | --- | --- |
| **Case** | **District** | **State** | **wy_ST_** | **wy_WA_** | **wy_AN_** | **wy_OW_** | **µ** | **σ** | **GM** | **LA** | **MANUSH  (α = 2)** |
| #1 | Dhar | Madhya Pradesh | 0.16 | 0.19 | 0.13 | 0.01 | 0.49 | 0.43 | 0.56 | 0.49 | 0.55 |
| #2 | Karnal | Haryana | 0.16 | 0.12 | 0.13 | 0.08 | 0.49 | 0.42 | 0.52 | 0.49 | 0.49 |

Here, it is also noted that only geometric mean and MANUSH method show change in their scores, thereby penalizing Dhar district for unbalanced development across a dimension of wasting and overweight, thus satisfying the condition of Uniformity. The linear aggregation method (LA), however, shows no change in its scores and does not penalise unbalanced development across dimensions.

Other instances where the condition of uniformity is being fulfilled is presented in Additional File 1.

**Shortfall Sensitivity.** *A measure of aggregation (LA, GM or MANUSH) should be such that for a given reduction in the aggregation value along its optimal path between two situations the reduction across dimensions should be in proportion to the shortfalls in the worse-off dimensions.*

If weighted standardized values in situation (#1) are such that wasting (q_1_) is greater than stunting (r_1_) which is greater than overweight/obesity (s_1_) which is greater than anemia (t_1_), or, q_1_> r_1_> s_1_> t_1_, then, in situation (#2) the reduction in failures should be such that (q_1_- q_2_) ≥ (q_1_/ r_1_) (r_1_- r_2_), (r_1_- r_2_) ≥ (r_1_/ s_1_) (s_1_- s_2_), and (s_1_- s_2_) ≥(s_1_/ t_1_) (t_1_- t_2_). We see situation 1a (Upper Siang in Arunanchal Pradesh) and situation 1b (Kollam in Kerala) in Table 1 mimic the same order of indicators, where wasting represents the worse-off dimension and anaemia represents the best-off dimension. Kollam when compared to Upper Siang, shows a lower reduction, in the worse off dimension, i.e. in wasting, when compared to anaemia, it's best off dimension, and hence is penalized by MANUSH measure (α ≥2). We even notice that when we increase α measure, that is α=3, 5 and 10, Kollam is further penalized on account of disproportionate reduction in its best -off and worse-off dimension. Conversely, when we study the situation 2a and 2b in table below, of Dakshin Dinajpur of West Bengal and Kanniyakumari of Tami Nadu, which follow the same order of indicator values, where, stunting represents the worse off dimension, followed by anaemia, wasting and overweight/obesity, represents the best-off dimension, we see that Kanniyakumari when compared to Dakshin Dinajpur, shows more reduction in its worse-off dimension, i.e. stunting, in comparison to its better off dimensions, it is not penalized by MANUSH measure (α ≥2), on account of being sensitive to shortfall across its best-off and worse-off dimension.

Table 5: Cases explaining the condition of Shortfall Sensitivity

|  | |  |  | Weighted Standardized values  (In descending order) | | | |  | | | | | |
| --- | --- | --- | --- | --- | --- | --- | --- | --- | --- | --- | --- | --- | --- |
| Situation | | District | State | q = wy_WA_ | r = wy_ST_ | s = wy_OW_ | t = wy_AN_ | q_1_-q_2_ | (q_1_/r_1_)*(r_1_-r_2_) | r_1_-r_2_ | (r_1_/s_1_)*(s_1_-s_2_) | (s_1_-s_2_) | (s_1_/t_1_)*(t_1_-t_2_) |
| 1 | a | Upper Siang | Arunanchal Pradesh | 0.18 | 0.09 | 0.08 | 0.07 | 0.06 | 0.07 | 0.04 | 0.03 | 0.03 | 0.05 |
|  | b | Kollam | Kerala | 0.11 | 0.06 | 0.05 | 0.03 |  |  |  |  |  |  |
| Situation | | District | State | q = wy_ST_ | r = wy_AN_ | s = wy_WA_ | t = wy_OW_ | q_1_-q_2_ | (q_1_/r_1_)*(r_1_-r_2_) | r_1_-r_2_ | (r_1_/s_1_)*(s_1_-s_2_) | (s_1_-s_2_) | (s_1_/t_1_)*(t_1_-t_2_) |
| 2 | a | Dakshin Dinajpur | West Bengal | 0.13 | 0.11 | 0.10 | 0.06 | 0.06 | 0.06 | 0.05 | 0.05 | 0.05 | 0.04 |
|  | b | Kanniyakumari | Tamil Nadu | 0.07 | 0.06 | 0.05 | 0.04 |  |  |  |  |  |  |

Table 5 continued

|  | |  |  | Aggregation Scores | | | | | |
| --- | --- | --- | --- | --- | --- | --- | --- | --- | --- |
| Situation | | District | State | GM | LA | MANUSH  (α = 2) | MANUSH  (α = 3) | MANUSH  (α = 5) | MANUSH  (α = 10) |
| 1 | a | Upper Siang | Arunanchal Pradesh | 0.439 | 0.426 | 0.447 | 0.470 | 0.510 | 0.559 |
|  | b | Kollam | Kerala | 0.255 | 0.251 | 0.273 | 0.294 | 0.325 | 0.357 |
| Situation | | District | State | State | LA | MANUSH  (α = 2) | MANUSH  (α = 3) | MANUSH  (α = 5) | MANUSH  (α = 10) |
| 2 | a | Dakshin Dinajpur | West Bengal | 0.427 | 0.401 | 0.404 | 0.406 | 0.409 | 0.413 |
|  | b | Kanniyakumari | Tamil Nadu | 0.226 | 0.221 | 0.220 | 0.220 | 0.220 | 0.220 |

This indicates that neither geometric mean nor linear aggregation methods are shortfall sensitive. Few more cases that explain shortfall sensitivity, and are reported in Additional File 1.

**Hiatus Sensitivity to Level**. *A measure of aggregation (LA, GM or MANUSH) should be such that the same gap (or hiatus) across dimensions should be considered worse-off as average failure decreases*.

For instance, if standardized values of stunting, wasting, anaemia and overweight/obesity are 0.18, 0.17, 0.13 and 0.03, respectively, in case #1 and 0.17, 0.16, 0.12 and 0.02, respectively, in case #2, as observed in Surendranagar of Gujarat and Bhojpur of Bihar, which has the same gap across dimension, then value of aggregation in case #1, in comparison to case #2, should have a lower deviation from its average failure (µ). We see that except MANUSH aggregation method, neither linear aggregation nor geometric mean, has a lower deviation from the average failure, in case of Surendranagar. Hence, the gap in MANUSH score between the two districts, i.e. Surendranagar and Bhojpur, are found to be narrow, compared to the gap in linear and geometric mean scores between two districts. Hence MANUSH is also hiatus sensitive. What this condition means, in essence, is that the gap between different dimensions should continue to reduce as a district moves towards a greater reduction in failure.

Table 6: Cases explaining the condition of Hiatus Sensitivity to Level

|  |  |  | **Weighted Standardized values** | | | | **Weighted Average** | **Aggregation Scores** | | | **ʋ_k_** | | |
| --- | --- | --- | --- | --- | --- | --- | --- | --- | --- | --- | --- | --- | --- |
| **Case** | **Districts** | **State** | **wy_ST_** | **wy_WA_** | **wy_AN_** | **wy_OW_** | **µ** | **GM** | **LA** | **MANUSH  (α = 2)** | **GM - µ** | **LA - µ** | **MANUSH - µ** |
| #1 | Surendranagar | Gujarat | 0.18 | 0.17 | 0.13 | 0.03 | 0.50 | 0.56 | 0.50 | 0.54 | 0.06 | 0.00 | 0.04 |
| #2 | Bhojpur | Bihar | 0.17 | 0.16 | 0.12 | 0.02 | 0.46 | 0.51 | 0.46 | 0.51 | 0.05 | 0.00 | 0.05 |

It is seen that all the three aggregation measures (Linear aggregation, Geometric Mean and MANUSH) satisfy the first three axioms while Geometric Mean satisfies additional condition of Uniformity. However, conditions of Shortfall Sensitivity and Hiatus Sensitivity to level is satisfied by MANUSH only, which makes it more robust compared to the other two aggregation measures.
